# The Prognostic Value of Pre-treatment Hemoglobin (Hb) in Patients With Advanced or Metastatic Gastric Cancer Treated With Immunotherapy

**DOI:** 10.3389/fonc.2021.655716

**Published:** 2021-06-15

**Authors:** Miaomiao Gou, Yong Zhang, Tiee Liu, Tongtong Qu, Haiyan Si, Zhikuan Wang, Huan Yan, Niansong Qian, Guanghai Dai

**Affiliations:** ^1^ Medical Oncology Department, The First Medical Center, Chinese People’s Liberation Army General Hospital, Beijing, China; ^2^ Medical Oncology Department, The Second Medical Center, Chinese People’s Liberation Army General Hospital, Beijing, China; ^3^ The Hainan Medical Center, Chinese People’s Liberation Army General Hospital, Sanya, China

**Keywords:** gastric cancer, immunotherapy, hemoglobin, prognostic bio-marker, PMS

## Abstract

**Background:**

Biomarkers such as prevailing PD-L1 expression and TMB have been proposed as a way of predicting the outcome of immunotherapy in patients with advanced gastric cancer (AGC) and metastatic gastric cancer (MGC). Our study aims to investigate whether there is a link between pretreatment hemoglobin (Hb) levels and survival to immunotherapy in patients with AGC and MGC.

**Methods:**

We retrospectively reviewed patients with AGC or MGC treated at the oncology department of the Chinese PLA general hospital receiving PD-1 inhibitor. The Propensity Score Matching (PSM) (1:1) was performed to balance potential baseline confounding factors. Progression-free survival (PFS) and overall survival (OS) was analyzed among different Hb level (normal Hb group and decreased Hb group). Objective response rate (ORR), disease control rate (DCR) were also analyzed. Univariate analysis and multivariate analysis were performed further to validate the prognostic value of Hb level.

**Results:**

We included 137 patients with AGC and MGC who received PD-1 inhibitors (including Pembrolizumab, Nivolumab, Sintilimab, Toripalimab) in this study. After PSM matching, there were no significant differences between the two groups for baseline characteristics. Within the matched cohort, the median PFS was 7.8 months in the normal Hb level group and 4.3 months in the decreased Hb group (HR 95% CI 0.5(0.31, 0.81), P=0.004). The OS was 14.4 months with normal Hb level as compared with 8.2 months with decreased Hb level(HR 95% CI 0.59(0.37, 0.94), P=0.024). The ORR was 40.7% and DCR was 83.0% in the normal Hb group, while the ORR was 25.5% and DCR was 85.1% in the decreased Hb group. No significant differences were found in the ORR and DCR between the two groups (P=0.127, P=0.779). Univariate analysis and multivariate analysis showed that Hb level was only independent predictor for PFS and baseline Hb level was significant prognostic factor influencing the OS. Only when patients had normal Hb level, anti-pd-1 monotherapy or combined with chemotherapy was superior to anti-pd-1 plus anti-angiogenic therapy with respect to PFS (10.3 m vs 2.8 m, HR 95% CI 0.37(0.15, 0.95), P=0.031) and OS(15 m vs 5.7 m, HR 95% CI 0.21 (0.08, 0.58), P=0.001).

**Conclusions:**

Our study have demonstrated that pretreatment Hb level was an independent prognostic biomarker in term of PFS and OS with immunotherapy for AGC and MGC patients. Correction of anemia for GC patients as immunotherapy would be a strategy to improve the survival. More data was warranted to further influence this finding.

## Introduction

Gastric cancer (GC) is the second most frequently diagnosed cancer and leading cause of cancer death in China, accounting for approximately 679.1 per 100,000 new cases (1). The majority of patients are diagnosed with locally advanced or metastatic disease, associated with poor median survivals (2). Up to now, the first-line treatment for advanced gastric cancer (AGC) or metastatic gastric cancer (MGC) with HER2-negative in Asia is either doublet regimen of S-1 or Capecitabine plus Cisplatin or Oxaliplatin (3). In the West, the first-line treatment is a three-drug combination regime such as docetaxel and cisplatin plus fluorouracil(DCF) (4). With both treatment regimes, the median survival time for patients with late-stage disease is still very low (less than 12 months) (4, 5).

The FDA has approved Nivolumab or Pembrolizumab, a fully human IgG4 monoclonal antibody targeting programmed death-1 (PD-1) as the third or further line of treatment for AGC or mGC, based on the Keynote 059 study (6) and ATTRICTION 2 trial (7). Pembrolizumab did not significantly improve overall survival compared with Paclitaxel as second-line therapy and for AGC with PD-L1 CPS of 1 or higher (8) and Pembrolizumab plus chemotherapy was not superior to chemotherapy for the OS and PFS as first-line therapy (9). However, anti-pd-1 therapy was still applied in clinic practice for AGC or mGC patients despite unsatisfactory trials results. As to this year, the data reported in ESMO from the ATTRACTION4 (10) and Checkmate 649 (11) have demonstrated encouraging efficacy with Nivolumab combined with SOX/CapeOX, irrespective of tumor PD-L1 expression. These results provide strong evidence for the use of immunotherapy for unresectable advanced or metastatic HER2-negative GC patients.

Blood-derived biomarkers have become a convenient and promising prognostic tool to identify and characterize the patients’ population. Some of the most used blood-derived biomarkers for GC are anemia (12), neutrophil-to-lymphocyte ratio(NLR) (13), platelet-to-lymphocyte ratio(PLR) (13), and tumor makers (14). Anemia occurs in most cancers patients and has multifactorial causes (15). Over the last decade, accumulating evidence has indicated that anemia is correlated with poor survival following chemotherapy in many types of cancers (16), including GC (12). However, the impact of hemoglobin (Hb) levels on prognosis in patients with AGC or MGC receiving immune therapy remains to be elucidated. Therefore, our study aims to investigate the prognostic value of pretreatment Hb level for patients who benefits from PD-1 inhibitor therapy with AGC and MGC.

## Patient And Method

### Study Population

A total of 197 patients with stage IV gastric cancer who received immune checkpoint inhibitor (ICI) treatment between October 1, 2015, and December 31, 2020 at Chinese PLA General Hospital were enrolled in this retrospective study. Patients who had unconfirmed pathology diagnose and had received anti-PD-L1 antibodies were excluded. Patients meeting the inclusion criteria were those who received at least two cycles of anti-PD-1, had measurable lesions. We also measured survival time and hemoglobin (Hb) levels before any line of immunotherapy. Clinical parameters including age, gender, PD-L1 expression status, Eastern Cooperative Oncology Group Performance Status (ECOG PS), tumor location, differentiation, undergone surgery, metastasis organs, personal history, therapy line, and regime were obtained from the patients’ #medical records. Based on these criteria, our study analyzed 137 gastric cancer patients. This study was approved by the Ethics Committee of Chinese PLA General Hospital and was conducted according to the principles of the Declaration of Helsinki.

### Treatment Regimens

The administrations of PD-1 inhibitor regimens consist of monotherapy or combined with chemotherapy or anti-angiogenic therapy. PD-1–targeting antibodies involved Nivolumab at a dose of 3 mg/kg, Pembrolizumab 200 mg intravenously, Sintilimab 200 mg per cycle, or Toripalimab at a stable 240 mg dosage with other treatment every 3 weeks. Chemotherapy regimens included XELOX (capecitabine 1,000 mg/m^2^ twice daily on days 1 to 14 of each cycle plus intravenous oxaliplatin 130 mg/m² on day 1 of each cycle), SOX (S-1 40–60 mg twice daily on days 1 to 14 plus oxaliplatin 130 mg/m^2^ on day 1) or DCF (docetaxel 75 mg/m^2^ cisplatin 75 mg/m^2^ on day 1 plus fluorouracil 750 mg/m^2^/d), and other combination. Angiogenesis inhibitors combined with those anti-checkpoint monoclonal antibodies are classified into small-molecule tyrosine kinase inhibitors (TKI) such as Apatinib and monoclonal antibody such as Bevacizumab. The regimen was based on the patients’ condition and preference. All patients signed informed consent for treatment.

### Assessment

In terms of baseline Hb levels, patients were divided into normal Hb group with level ≥110 g/L and decreased Hb group at level <110g/L, according to the National Comprehensive Cancer Network (NCCN)guideline. The tumor response through the whole cycle included complete response (CR), partial response (PR), stable disease (SD), and progressive disease (PD). The response was evaluated according to Response Evaluation Criteria in Solid Tumors 1.1 (17). The objective response rate (ORR) was defined as the percentage of patients who achieved a CR or PR, and the disease control rate (DCR) was the percentage of patients with a CR or PR or SD. Overall survival (OS) was defined as the duration from the time of treatment to the time of death, and Progression-free survival (PFS) was defined as the time from treatment to the first occurrence of PD, or death.

### Statistical Analysis

Baseline characteristics were presented as proportions for categorical variables, which were compared using the Chi-square test. Propensity score-matched analysis was performed to control for confounding factors. The propensity score (1:1) was estimated using non-parsimonious multivariate logistic regression according to the following variables: age, gender, PD-L1 expression status, Eastern Cooperative Oncology Group Performance Status(ECOG PS), tumor location, differentiation, undergone surgery, metastasis organs, personal history, therapy line and regime. The Kaplan–Meier method was used to analyze survival data. The Log-rank test and Cox proportional hazard regression were performed to assess the association of Hb levels with OS and PFS and other clinical features. P < 0.05 denoted statistical significance in all analyses. All data were analyzed by SPSS 21.0 statistical software (SPSS Inc., Chicago, IL, USA).

## Results

A total of 137 patients with AGC and MGC received anti-PD-1 treatment at the Chinese PLA General Hospital during the study period. Baseline demographic characteristics including gender, age, PD-L1 expression status, ECOG PS, tumor location, histology differentiation, prior surgery, metastasis organs, smoking and drinking history, therapy line, and regime grouped by Hb Level (normal Hb level or decreased Hb level) is summarized in **Table 1**. Before propensity score matching, in contrast with decreased Hb level group, normal Hb group had higher rate of male proportion (78.2% vs 62.7%, p=0.047) and ECOG 0-1 patients (93.6% vs 83.1%, p=0.046). After propensity score matching (1:1), 47 patients in each normal Hb level group and decreased Hb level group were enrolled. No differences were observed between the two groups in terms of gender and ECOG PS score, and other variables. Thus, the two groups were well balanced for further comparison.

**Table 1:** Baseline demographic characteristics of patients with AGC or MGC by Hb level before and after propensity score matching.

Characteristics	Before propensity score matching					After propensity score matching				
	Hb<110 g/L	%	Hb>=110 g/L	%	P	Hb<110 g/L	%	Hb>=110 g/L	%	P
No. patients	59		78			47		47		
Gender n (%)										
Male	37	62.7%	61	78.2%	0.047	31	66.0%	35	74.5%	0.370
Female	22	37.3%	17	21.8%		16	34.0%	12	25.5%	
Age median =59 n (%)										
<59	28	47.5%	28	35.9%	0.174	20	42.6%	23	48.9%	0.537
>=59	31	52.5%	50	64.1%		27	57.4%	24	51.1%	
PD-L1 n (%)										
Positive	15	25.4%	14	17.9%	0.282	8	17.0%	5	10.6%	0.343
Negative	11	18.6%	14	17.9%		10	21.3%	9	19.1%	
Unknown	33	55.9%	50	64.1%		29	61.7%	33	70.2%	
ECOG PS n (%)										
0	12	20.3%	24	30.8%	0.046	12	25.5%	10	21.3%	0.957
1	37	62.7%	49	62.8%		29	61.7%	33	70.2%	
>=2	10	16.9%	5	6.4%		6	12.8%	4	8.5%	
Tumor_location n (%)										
Cardia	14	23.7%	25	32.1%	0.223	13	27.7%	10	21.3%	0.443
Body/Fundus	39	66.1%	48	61.5%		31	66.0%	33	70.2%	
Pylorus	6	10.2%	5	6.4%		3	6.4%	4	8.5%	
Histological_differentiation n (%)										
Poorly	32	54.2%	39	50.0%	0.695	22	46.8%	23	48.9%	0.847
Moderately	24	40.7%	36	46.2%		23	48.9%	22	46.8%	
Well	3	5.1%	3	3.8%		2	4.3%	2	4.3%	
Surgery n (%)										
Yes	27	45.8%	39	50.0%	0.624	21	44.7%	23	48.9%	0.681
No	32	54.2%	39	50.0%		26	55.3%	24	51.1%	
No. of metastasis organs n (%)										
<=2	38	64.4%	53	67.9%	0.665	34	72.3%	32	68.1%	0.654
>2	21	35.6%	25	32.1%		13	27.7%	15	31.9%	
Liver metastasis n (%)										
Yes	19	32.2%	38	48.7%	0.053	18	38.3%	17	36.2%	0.832
No	40	67.8%	40	51.3%		29	61.7%	30	63.8%	
Smoking_history n (%)										
Yes	21	35.6%	36	46.2%	0.216	19	40.4%	21	44.7%	0.678
No	38	64.4%	42	53.8%		28	59.6%	26	55.3%	
Drinking_history n (%)										
Yes	22	37.3%	37	47.4%	0.237	21	44.7%	24	51.1%	0.538
No	37	62.7%	41	52.6%		26	55.3%	23	48.9%	
ICIs therapy line n (%)										
First line	28	47.5%	38	48.7%	0.829	24	51.1%	25	53.2%	0.924
Second line	30	50.8%	34	43.6%		23	48.9%	19	40.4%	
Third line	1	1.7%	6	7.7%		0	0.0%	3	6.4%	
Treatment type n (%)										
ICIs monotherapy	10	16.9%	5	6.4%	0.054	5	10.6%	5	10.6%	0.837
Combination therapy										
ICIs plus chemotherapy	39	66.1%	53	67.9%		34	72.3%	35	74.5%	
ICIs plus anti-angiogenic therapy	10	16.9%	20	25.6%		8	17.0%	7	14.9%	

**Figure 1:**
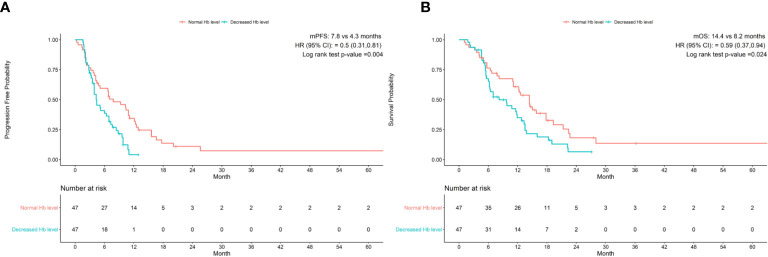
Baseline Hb levels associated with **(A)** progression-free survival and **(B)** overall survival in patients treated with anti-pd-1 inhibitors. CI, confidence interval; Hb, hemoglobin; HR, hazard ratio; mOS, median overall survival; mPFS, median progression-free survival.

After matching, the PFS was 7.8 months in the normal Hb level group and 4.3 months in the decreased Hb group with immunotherapy for GC patients. The OS was 14.4 months with normal Hb level as compared with 8.2 months with decreased Hb level. There were significant differences in PFS and OS between two groups (HR 95% CI 0.5(0.31, 0.81), P=0.004, HR 95% CI 0.59(0.37, 0.94), P=0.024, respectively) (**Figure 1**).

The response is presented in **Table 2**. After matching, an overall response was observed in 83.0% of the patients in the normal Hb level set and 85.1% in the decreased Hb group, with complete responses in 3 patients (6.3%) and 0 (0%), respectively. We recorded an objective response (ORR) in 19 of 47 patients (40.4%) in the normal Hb level patients and 12 out of 47 patients (25.5%) in the counterpart group. However, no statistical differences were found in ORR and DCR between the two groups (p=0.127, p=0.779, respectively).

**Table 2:** The confirmed response of patients with AGC or MGC by Hb level before and after propensity score matching.

Response	Before propensity score matching					After propensity score matching				
	Hb<110 g/L	%	Hb>=110 g/L	%	P	Hb<110 g/L	%	Hb>=110 g/L	%	P
No. patients	59		78			47		47		
Response										
CR	0		3		0.126	0		3		0.245
PR	15		27			12		16		
SD	33		36			28		20		
PD	11		12			7		8		
ORR					0.109					0.127
CR+PR	15	25.4%	30	38.5%		12	25.5%	19	40.4%	
DCR					0.615					0.779
CR+PR+SD	48	81.4%	66	84.6%		40	85.1%	39	83.0%	

**Figure 2:**
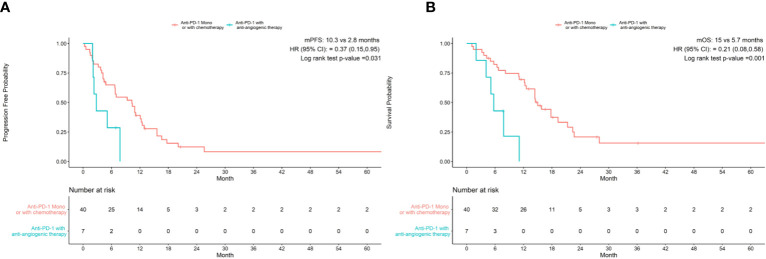
Treatment type associated with progression-free survival **(A)** and overall survival **(B)** in patients with normal Hb levels.

**Figure 3:**
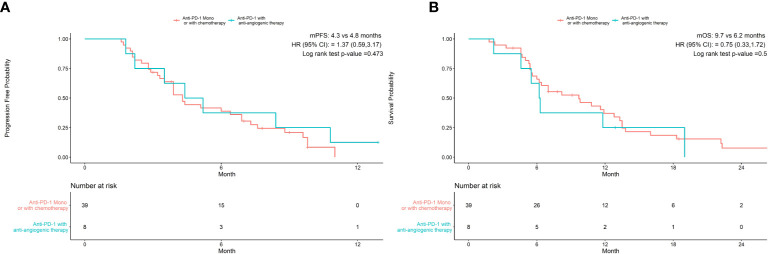
Treatment type hadn’t associated with progression-free survival **(A)** and overall survival **(B)** in patients with decreased Hb levels.

As shown in **Table 3**, univariate analysis revealed that Hb level and ECOG PS were significantly associated with PFS (P=0.004, P=0.044, respectively). Multivariate analysis was consistent with the outcomes that Hb level was the only independent predictor for PFS. On the other hand, in the univariate analysis for OS, histological differentiation, therapy line, treatment type, baseline Hb level were all related to overall survival time. Only baseline Hb level was a significant prognostic factor influencing the OS (**Table 4**). We further performed a log-rank test in different Hb level groups to demonstrate whether Hb level had an impact on anti-PD-1 based treatment. Interestingly, anti-PD-1 monotherapy or combined with chemotherapy was superior to anti-PD-1 plus anti-angiogenic therapy with respect to PFS [10.3m vs. 2.8m, HR 95% CI 0.37 (0.15, 0.95), P=0.031] and OS[15m vs. 5.7m, HR 95% CI 0.21 (0.08, 0.58), P=0.001] in normal Hb level group (**Figure 2**). In contrast, there was no evidence consistent with the above finding in decreased Hb level group (P>0.05) (**Figure 3**).

**Table 3:** Univariate analysis and multivariate analysis of clinical variable for the prediction of progression free survival of AGC patients treat with anti-pd-1 inhibitors.

Variable Category	Category	Univariate analysis		Multivariate analysis	
		HR (95% CI)	p-value	HR (95% CI)	p-value
		HR (95% CI)	p-value	HR (95% CI)	p-value
Gender	Female versus Male	0.97(0.59–1.61)	0.929	0.54(0.28–1.04)	0.609
Age	≧̸59 versus <59	1.17(0.74–1.84)	0.468	0.87(0.50–1.49)	0.613
ECOG	≧̸2 versus 0-1	1.80(0.94–3.46)	0.044	2.04(0.96–4.33)	0.062
Tumor_location	Cardia versus Body/Fundus versus Pylorus	0.87(0.60–1.27)	0.511	0.99(0.65–1.53)	0.995
Histological_differentiation	Poorly versus Moderately versus Well	0.71(0.47–1.05)	0.127	0.59(0.35–1.04)	0.051
Surgery n	Yes verse No	0.865(0.55–1.34)	0.516	1.09(0.65–1.85)	0.744
No.of metastasis organs	>2 verse <=2	1.47(0.92–2.36)	0.1	1.35(0.77–2.35)	0.289
Liver metastasis	Yes verse No	1.0(0.63–1.59)	0.993	1.09(0.63–1.89)	0.743
Smoking_history	Yes verse No	1.23(0.79–1.91)	0.348	1.08(0.58–2.01)	0.802
Drinking_history	Yes verse No	0.85(0.53–1.33)	0.486	0.62(0.32–1.19)	0.154
ICIs therapy line	Second and Third line verse First line	1.46(0.94–2.26)	0.086	1.26(0.74–2.12)	0.384
Treatment type	Anti-pd-1 plus anti-angiogenic therapy verse	1.49(0.81–2.72)	0.187	1.26(0.60–2.64)	0.536
	Anti-pd-1 monotherapy or with chemotherapy				
Baseline Hb, g/L	≧̸110 versus <110	0.50(0.31–0.81)	0.004	0.46(0.26–0.82)	0.008

CI, confidence interval; ECOG PS, Eastern Cooperative Oncology Group Performance Status; Hb, hemoglobin; HR, hazard ratio.

**Table 4:** Univariate analysis and multivariate analysis of clinical variable for the prediction of overall survival of AGC patients treat with anti-pd-1 inhibitors.

Variable Category	Category	Univariate analysis		Multivariate analysis	
		HR (95% CI)	p-value	HR (95% CI)	p-value
Gender	Female versus male	0.92(0.54–1.66)	0.76	0.46(0.23–0.93)	0.024
Age	≧̸59 versus <59	1.07(0.67–1.70)	0.645	0.88(0.51–1.53)	0.655
ECOG	≧̸2 versus 0-1	1.04(0.51–2.12)	0.667	1.41(0.60–3.31)	0.42
Tumor_location	Cardia versus body/fundus versus pylorus	0.88(0.59–1.33)	0.509	1.04(0.65–1.67)	0.844
Histological_differentiation	Poorly versus moderately versus well	0.63(0.41–0.98)	0.042	0.60(0.36–1.01)	0.059
Surgery	Yes verse No	0.69(0.43-1.11)	0.125	0.69(0.40–1.19)	0.188
No.of metastasis organs	>2 verse <=2	1.61(0.99–2.62)	0.049	1.07(0.60–1.91)	0.803
Liver metastasis	Yes verse No	0.89(0.55–1.44)	0.658	0.88(0.50–1.53)	0.656
Smoking_history	Yes verse No	1.19(0.75–1.89)	0.453	1.10(0.56–2.17)	0.772
Drinking_history	Yes verse No	0.81(0.50–1.29)	0.371	0.56(0.27–1.13)	0.107
ICIs therapy line	Second and third line versus first line	1.76(1.11–2.80)	0.014	1.71(0.97–3.02)	0.063
Treatment type	Anti-pd-1 plus anti-angiogenic therapy versus Anti-pd-1 monotherapy or with chemotherapy	2.28(1.23–4.25)	0.007	2.1(0.95–4.63)	0.064
Baseline Hb, g/L	≧̸110 versus <110	0.59(0.37–0.94)	0.024	0.51(0.29–0.89)	0.018

CI, confidence interval; ECOG PS, Eastern Cooperative Oncology Group Performance Status; Hb, hemoglobin; HR, hazard ratio.

## Discussion

In the era of immunotherapy, immune checkpoint inhibitors (ICI) are showing great benefits for late stage cancers, such as lung cancer (18–21), esophageal cancer (20, 22), melanoma cancer (23), renal cell cancer (24). Studies on immunotherapy for gastric cancer, on the other hand, have been controversial. It appears that Nivolumab and Pembrolizumab have a different outcome for patients with advanced GC when combined with chemotherapy or not in the first or second-line (8–10). The underlying reason still remain unclear. Well-known biomarker surrounded with PD-L1 expression and tumor mutation burdens (TMB) beside MSI do not fully explain why immunotherapy did not improve the survival compared with standard treatment (9, 25). Thus, additional factors or biomarkers are needed to predict benefit for patients.

To some extent, laboratory-based biomarkers such as anemia, NLR, PLR, and dynamic tumor makers before or after treatment had demonstrated a predictor value for anti-cancer therapy in several tumors. While some data suggest that anemia is not an important prognostic factor, a large number of prospective and retrospective studies, as well as data from *in vitro* and *in vivo* studies, suggest that Hb levels may influence treatment outcome parameters (26–28). To our knowledge, however, no study had yet investigated the Hb level as a prognostic maker for patients undergoing immunotherapy.

Our analysis indicated that normal pre-treatment Hb level was a significant predictor when associated with higher PFS and OS in patients receiving anti-PD-1 therapy, as compared with decreased Hb level. This result suggest that patients with AGC and MGC suffering anemia might need more consideration of immunotherapy. Patients with normal Hb level receiving PD-1 inhibitor were shown to have prolonged long term benefit of 14.4months and progression time of 7.8m (p<0.05). This was in line with previous studies of NSCLC treated with ICI (29, 30). Hypoxia is a potent barrier to effective immunotherapy in cancer treatment (31). The reason could be multifactorial and remain to be addressed. Anemia is common in cancer patients and is suspected to contribute to intratumoral hypoxia (32). Hypoxia induced by anemia has been proved to lead in tumor growth and progression and decrease their sensitivity to anticancer treatments (33). In addition, Zhao L et al. (34) observed that anemia is also associated with T-cell deficiency in a mouse model. T cell play an important role on tumor microenviroment and anticancer process (35) as we know. Therefore, anemia may attenuate the T-cell surrounding the tumor. These findings might explain why immunotherapy was less effective in patients with lower levels of Hb. This conclusion suggests that correcting anemia in GC patients undergoing immunotherapy could bring benefits for their survival.

Regarding the response rate, there were no difference of DCR or ORR for GC patients with and without anemia. It appears that anemia does not impact tumor shrinkage when using PD-1 inhibitor alone or combined with other cytotoxicity drug. This is consistent with the result of a study conducted by Qing Wei et al. (12). This study showed no difference in chemotherapy outcome between patients with and without anemia regardless of regional and racial differences and different definitions of anemia. However, these results will require further study to better elucidate the role of anemia.

Investigation of whether Hb level was independent prognostic indictor for PFS and OS, univariate analysis and multivariate analysis were performed and the data indicated patients with anemia was poor predictor for both PFS and OS. The result further added evidence that Hb level was a potent biomarker for survival benefit population. Interestingly, we also found that patients with normal pre-treatment Hb levels treated with anti-PD-1 treatment alone or with chemotherapy achieved longer PFS (10.3 m vs 2.8 m, p<0.05) and OS (15 m vs 5.7 m, p<0.05) than those receiving anti-PD-1 plus anti-angiogenic therapy. The same outcome was not obtained from cases with decreased baseline Hb levels (PFS 4.3 m vs 4.8 m, p>0.05) and (OS 9.7 m vs 6.2 m). In the REGONIVO study (36), the combination of Regorafenib (an oral multikinase inhibitor for angiogenesis) plus Nivolumab had an encouraging antitumor activity (ORR=44%) in patients with gastric cancer after failure to prior treatment. Therefore, the addition of anti-angiogenic monoclonal antibodies or TKI to immunotherapy for advanced gastric cancer might be an alternative therapeutic options for these patients. The data suggest that strategies targeting angiogenesis with ICI are more suitable for patients with anemia than normal Hb status. This might be attributed to antiangiogenic agents may transiently normalize blood vessels and decrease hypoxia (37), which plays a bigger role in the presence of anemia. This in turn may also help us to make best treatment options for patient with advanced GC in different situation.

This study had several limitations. This report was a single-center retrospective study with a limited sample size and possible incomplete information. Due to limited known status of PD-L1 expression, no correlation between PD-L1 expression and survival was noted in this study. MorA bigger population study would be needed to verify the results of the study.

## Conclusion

Our study demonstrates that pretreatment Hb levels are associated with PFS and OS for immunotherapy with patients of AGC and MGC. Anemia is a potential prognostic biomarker. Larger samples are needed to further validate its predictive value.

## Data Availability Statement

The raw data supporting the conclusions of this article will be made available by the authors, without undue reservation.

## Ethics Statement

This study was approved by the Ethics Committee of Chinese PLA General Hospital and was conducted according to the principles of the Declaration of Helsinki.

## Author Contributions

MG and YZ were in charge of writing and analysis. TL, TQ, HS, ZW, and HY provided the data. NQ and GD provided the guide and idea. All authors contributed to the article and approved the submitted version.

## Funding

This study was supported by the National Natural Science Foundation of China (no.31671298).

## Conflict of Interest

The authors declare that the research was conducted in the absence of any commercial or financial relationships that could be construed as a potential conflict of interest.
